# Population genomic analysis identifies the complex structural variation at the fibromelanosis (*FM*) locus in chicken

**DOI:** 10.1038/s41598-025-94250-4

**Published:** 2025-03-18

**Authors:** Cheng Ma, Leif Andersson

**Affiliations:** 1 Department of Medical Biochemistry and Microbiology, Uppsala University, Uppsala, Sweden; 2 Department of Veterinary Integrative Biosciences, Texas A&M University, College Station, USA

**Keywords:** Structural variation, Population genetics, Agricultural genetics

## Abstract

Phenotypic diversity and its genetic basis are central questions in biology, with domesticated animals offering valuable insights due to their rapid evolution the last 10,000 years. In chickens, fibromelanosis (FM) is a striking pigmentation phenotype characterized by hyperpigmentation. A previous study identified a complex structural variant involving both two large duplications (127.4 and 170.5 kb in size) and inversions associated with upregulated expression of the *Endothelin 3* (*EDN3*) gene. However, the detailed organization of the structural arrangements have remained unclear. In this study, we conducted a comprehensive genomic survey of 517 FM chickens representing 44 different populations. Our results elucidate the complex arrangement of the duplications and inversions at the *FM* locus based on the large-scale genomic survey, population level genotyping, and linkage disequilibrium analysis, providing conclusive support for one specific configuration of the two large duplications, resolving a controversy that has been unresolved for more than a decade. Our results show that the birth of this complex structural variant must have involved an interchromosomal rearrangement creating fixed heterozygosity due to sequence differences between the two copies of the 127.4 kb duplication. This study shows how population genomics can be used to understand complex structural variations that underlie phenotypic variation.

## Introduction

How phenotypic diversity evolves and its genetic basis is a fundamental question in biology. Domesticated animals constitute a valuable resource to explore this topic due to their rapid phenotypic evolution within the last 10,000 years^1^. A characteristic feature of domestic animals is altered pigmentation pattern, a phenotypic change that occurred early during domestications of animals as documented by ancient illustrations and documents. A striking pigmentation phenotype in domestic chicken is fibromelanosis (FM) that is widespread among Asian chicken breeds, like Chinese Silkie and Ayam Cemani from Indonesia. FM is characterized by a massive expansion of melanocytes resulting in excessive skin and tissue pigmentation^2^. The FM trait is highly valued culturally and commercially in Asia. For instance, Silkie chickens are prized in ornamental breeding for their distinctive appearance, while Ayam Cemani chickens hold cultural significance and command high market prices^3^. FM chickens are valued for their deep eumelanin deposition, used in food such as Chinese black-bone soup and in Chinese traditional medicine^4,5^.

**Fig. 1:**
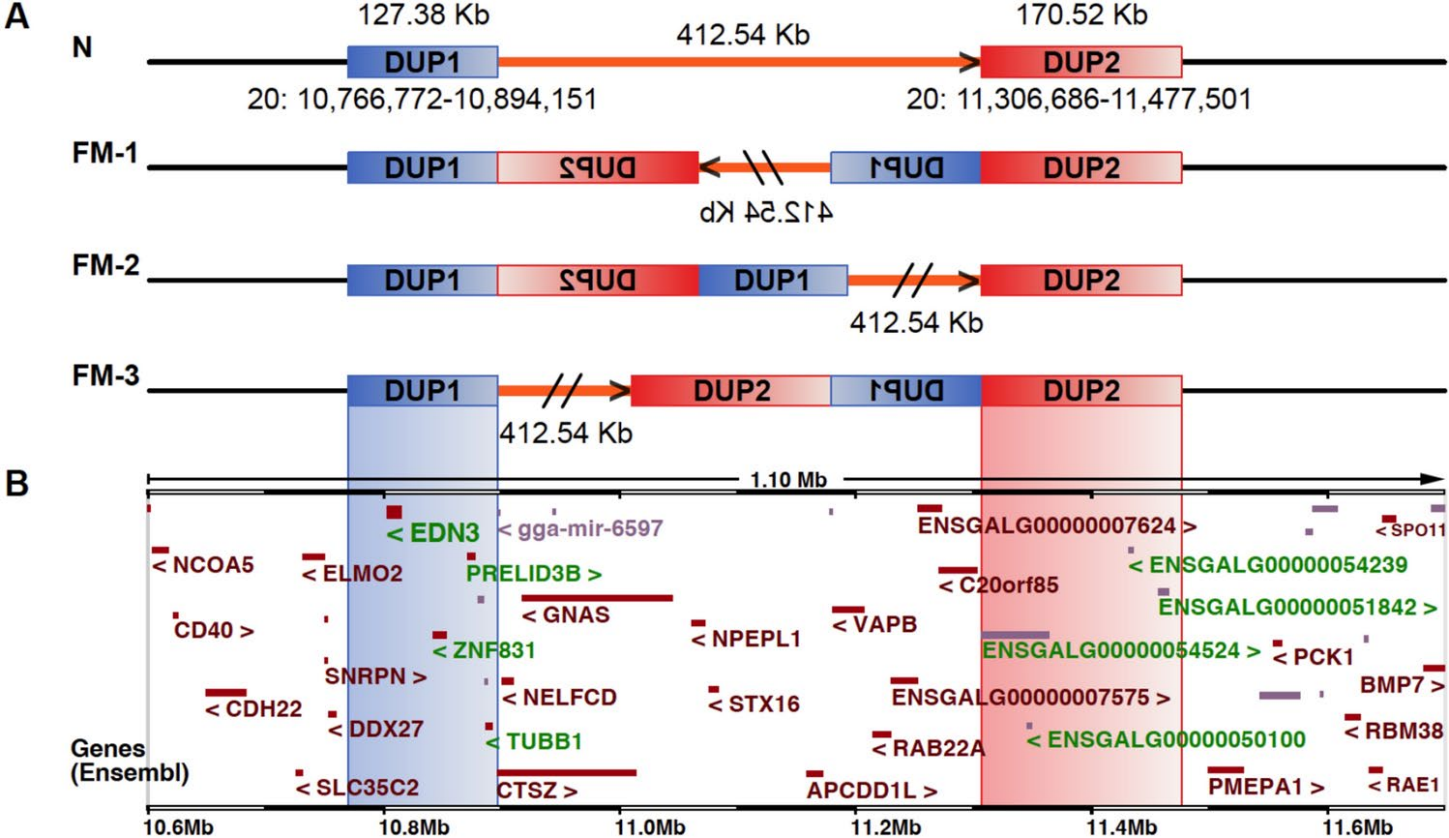
Possible structural variant arrangements at the *FM* locus in chickens. (**A**) Schematic representation of the wild type and three candidate arrangements of SVs in FM chickens^2^ from 10.6 to 11.7 Mb of chromosome 20. The genomic regions involved are illustrated with different colors: DUP1 in blue, DUP2 in red, and the intermediate region in orange. Orientations of the genome sequence are indicated with arrows and linear gradient colors. The wild type arrangement at the top shows no structural variations in the FM region. FM-1 implies an inversion of an entire block including duplicated copies of DUP1 and DUP2, together with the non-duplicated region between DUP1 and DUP2. FM-2 and FM-3 involve inversions of only DUP2 or DUP1, respectively. (**B**) Genomic annotation of the target region shown in (**A**). Ensembl protein-coding genes are shown in dark brown, RNA genes in light purple, and genes within DUP1 and DUP2 are highlighted in green. The two duplications are marked by shading: DUP1 in blue and DUP2 in red.

A previous study demonstrated that the dominant FM trait in several breeds of chicken is caused by a complex structural rearrangement involving two duplications, 127.4 and 170.5 kb in size^2^. One of the duplications encompass the *EDN3* gene, which has a critical role for melanoblast differentiation and expansion^6^. The fact that *EDN3* shows a highly significant upregulation at the mRNA level in skin from FM chickens strongly suggested that this is the causal mechanism for the massive expansion of pigment cells in FM chicken^2^. It was not possible to resolve the organization of the complex rearrangement with the short-read whole genome sequence data available a decade ago, even with the structural variation detection tools widely used today^7^, and three possible configurations of the complex structural rearrangement (Fig. 1) were established based on PCR analysis of breakpoint regions^2^. A single recombinant found in a pedigree segregating for the *FM* mutation was only consistent with one of the possible configurations denoted FM-2^2^. However, more recent data based on long read sequencing assemblies have given conflicting results, in which some studies^2,8,9^ have supported the FM-2 configuration whereas other studies came to the conclusion that their assembly of this region from Yeonsan Ogye^10^ and Silkie^11^ chicken supported the FM-1 configuration.

**Fig. 2:**
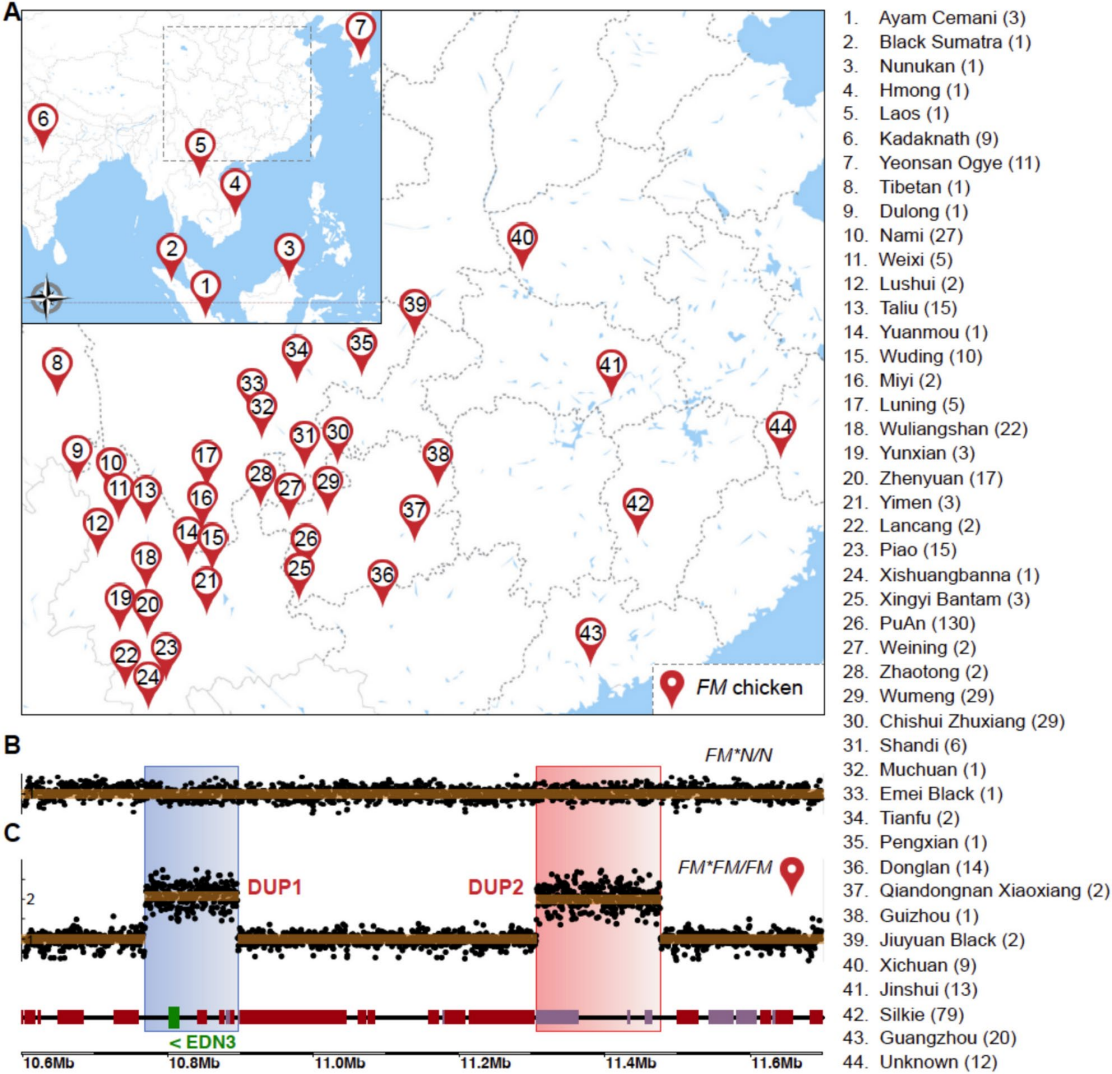
Characterization of structural variants associated with fibromelanosis (*FM*) in chickens. (**A**) Geographic distribution and breed representation of the 517 FM chickens used in this study, representing 44 breeds. The area within the dotted box is shown in an enlarged view. The number in each icon corresponds to the breeds listed on the right, with the number in brackets indicating the sample size for each breed. (**B**, **C**) Relative sequence depth revealing the genomic locations of the two different duplications identified in FM chickens; DUP1 is marked in blue, and DUP2 is marked in red. The genomic annotations of these regions are shown at the bottom of panel (**C**). The *EDN3* gene is high-lighted in green. (**B**) Wild type (FM*N/*N*). (**C**) *FM* homozygote (FM*FM/*FM*).

In order to resolve the structural arrangement of the *FM* locus, here we have analyzed whole genome sequence data from 517 FM chickens representing 44 chicken breeds worldwide, all with the FM phenotype (Fig. 2A). Our comprehensive population genomic analysis showed that all these breeds have the same structural variations (SVs) at the *FM* locus as previously reported^2^. Our population genomic analysis provides overwhelming support for the FM-2 model based on the pattern of linkage disequilibrium across the complex rearrangement reflecting where recombination events can occur. This study highlights how population genomics data can be used to resolve complex structural variations that are challenging to resolve even using long-read sequence data.

## Results

### Comprehensive genomic survey of the *FM* locus

We analysed whole genome sequencing data of 517 FM chickens representing 44 breeds of chicken (Fig. 2A). Our results showed that all these breeds have the same structural variants (SVs) at the *FM* locus (Fig. 2B and C). The SVs involve duplications of two genomic regions on chromosome 20:10,766,772–10,894,151 bp (DUP1) and chr20:11,306,686–11,477,501 bp (DUP2) (Fig. 2C), consistent with previous findings^2,12^.

### Genetic profile of the *FM* allele

**Fig. 3:**
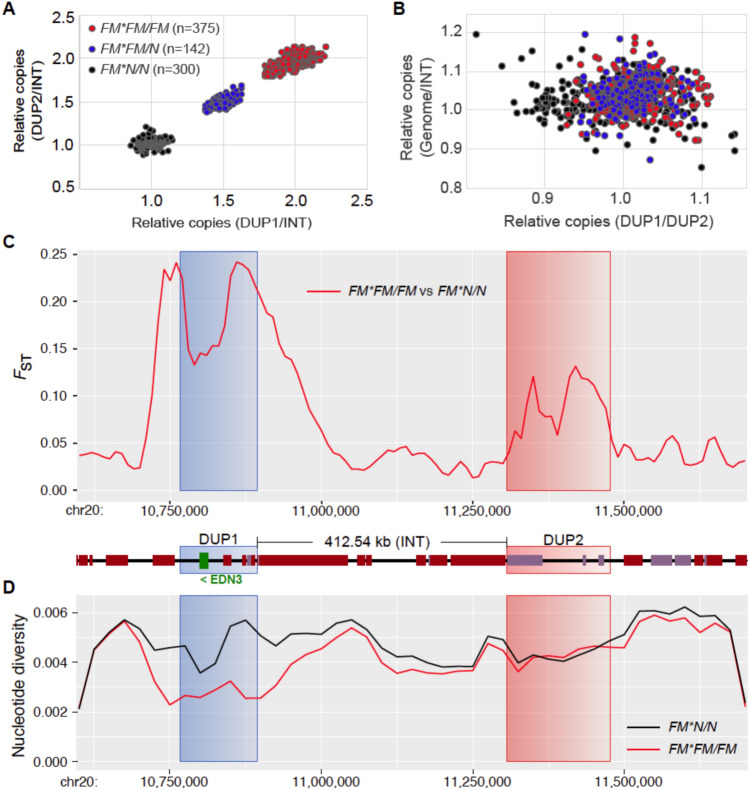
Relative sequencing depth and nucleotide diversity at the *FM* locus. (**A**, **B**) Relative sequence depth analysis was used to deduce wild type (FM*N/*N*), *FM* homozygous (FM*FM/*FM*), and *FM* heterozygous (FM*FM/*N*) samples. (**A**) Relative copy number of the DUP1 and DUP2 regions compared with the intermediate (INT) region. *FM* homozygous samples are in red, *FM* heterozygous samples are in blue, and wild type samples are in black. The colors of the different points are indicated in the legend, and the number in brackets in the legend indicates the corresponding sample size. (**B**) Relative sequence coverage of DUP1/DUP2 compared with sequence coverage of the INT region compared with genome-wide coverage in all samples. The color code is the same as in (**A**). (**C**) Genetic differentiation measured using *F*_ST_ between *FM* homozygotes and wild type homozygotes. Genetic differentiation was significantly higher at the *FM* locus compared to whole genome average background (average *Z* = 13.05, *P* = 1.44e-5 for DUP1; average *Z* = 5.72, *P* = 0.011 for DUP2). (**D**) Nucleotide diversity plot for *FM* homozygotes and wild type groups across the FM region. The FM duplicated regions are marked using the same background colors as above.

**Fig. 4:**
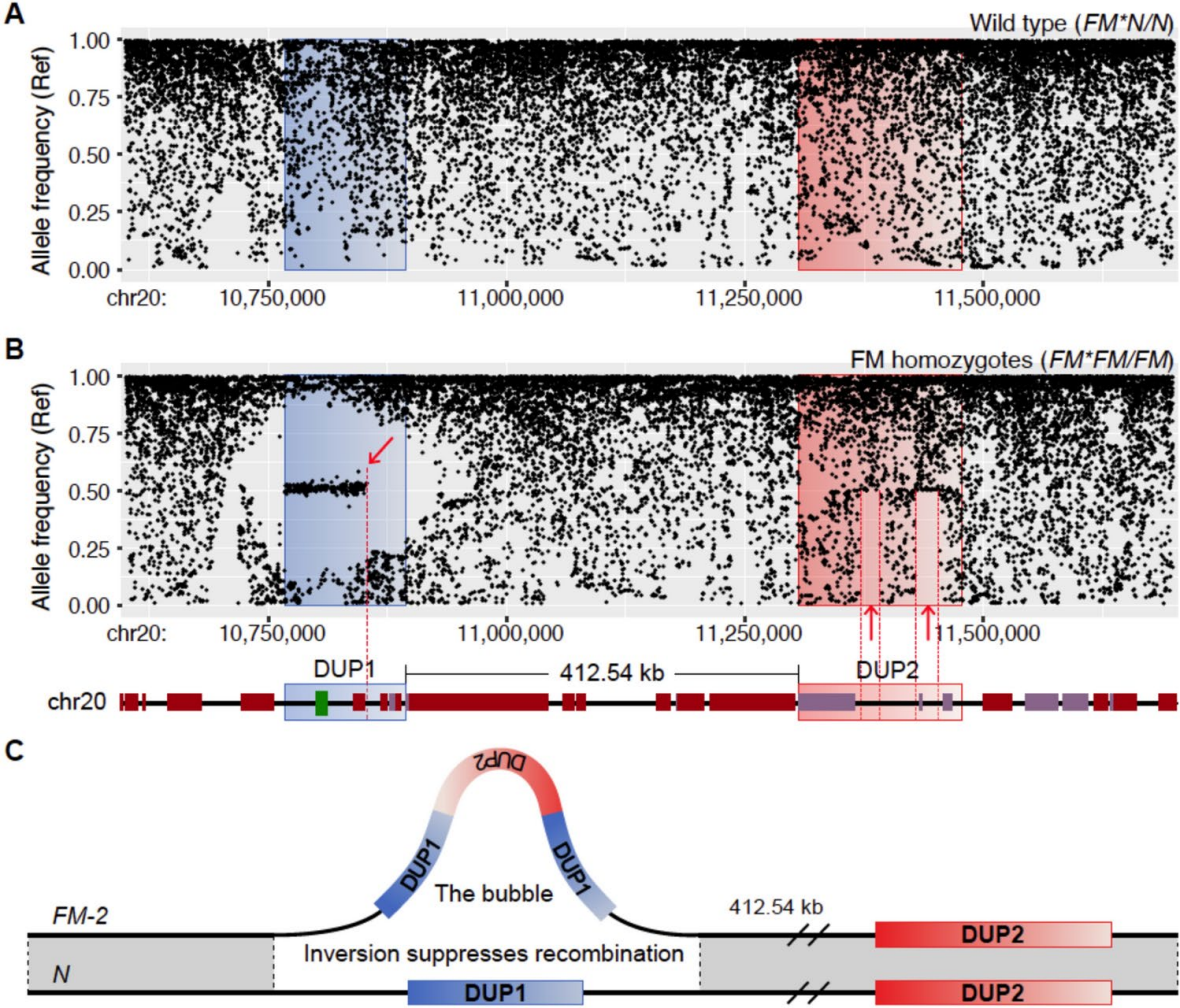
Allele frequency patterns and structure at the *FM* locus in chickens. (**A**) Frequency of the reference allele in wild type (FM*N/*N*) chickens across the FM region. The regions analyzed include DUP1 (blue) and DUP2 (red), with their relative positions and orientations indicated. (**B**) Allele frequency distribution in homozygous FM chickens (FM*FM/*FM*), showing a distinct bubble covering the DUP1 region, indicating strong suppression of recombination. A region with fixed heterozygosity is notable, showing an allele frequency of 0.5 for the reference allele. A breakpoint in the region of fixed heterozygosity is noted and highlighted with a red arrow. Two reference-consistent segments in DUP2 are shown with red dotted box and arrows. Schematic annotation of the genomic regions analyzed for allele frequency patterns. Ensembl protein-coding genes are shown in dark brown, RNA genes in light purple. (**C**) Diagram showing the suppression of recombination between *FM* and *WT* allele in the DUP1 region. The suppression is results from the inversion of DUP2.

We performed a comprehensive analysis of genomic data to characterize the arrangement of the complex chromosomal structural variation of the *FM* allele (Figs. 3 and 4). Firstly, we analyzed the sequence depth at the two duplications associated with the FM phenotype and confirmed that DUP1 and DUP2 occur as two copies in the *FM* allele (Fig. 3A and B). This analysis allowed us to identify *FM* homozygotes that were used in the further analysis. We next determined the genetic differentiation between wild-type (FM*N/*N*) and *FM* homozygous chickens (FM*FM/*FM*) (Fig. 3C). *F*_ST_ results showed that there were significant genetic differences in the two SV regions, especially in DUP1 and its flanks. Notably, there is an obvious reduction of genetic differentiation at the front segment of the DUP1 region (Fig. 3C). Nucleotide diversity analysis showed a marked reduction of heterozygosity in *FM* homozygotes but interestingly only in the DUP1 region and its flanks (Fig. 3D).

We next analyzed the frequency of the reference allele for all SNPs across the region harboring the two duplications. This revealed a remarkable bubble for the DUP1 region in *FM* homozygotes not present in wild-type chicken (Fig. 4A and B). In the bubble region, *FM* homozygotes tended to be fixed for the reference allele or the non-reference allele, or the two alleles occur at exactly the same frequency (50%) (Fig. 4B). A weak tendency for a similar pattern was noted for the DUP2 region where two regions (marked with red arrows) had an excess of SNPs showing an allele frequency of 50% (Fig. 4B). The non-duplicated 412.54 kb region between DUP1 and DUP2 showed a very similar distribution of allele frequencies as present in wild-type chickens (Fig. 4A and B). These data are consistent with strong suppression of recombination between FM and wild-type chromosomes but only for the DUP1 region.

### Characterization of the structural arrangement of *FM* allele

The above results refute FM-1 as a possible organization of the *FM* allele. This is because the FM-1 configuration (Fig. 1A) implies an inversion involving one copy of DUP1, the connecting region (412.54 kb) and one copy of DUP2, and the bubble should extend across the entire region which it does not (Fig. 4B). We therefore conclude that the FM-2 configuration must be the correct organization because it is the only one consistent with a single previously reported recombination event^2^ and none of the recent reports based on PacBio long reads found support for the FM-3 order^10,11^. In addition, if the configuration of FM-3 is correct, we should observe a bubble in the DUP2 region, not in the DUP1 region. Phylogenetic trees constructed for the DUP1 and DUP2 regions revealed differences in branch lengths between two SV regions which support the hypothesis of an inversion near the DUP1 region (Supplementary Fig. 1), consistent with our proposed structural configuration of the *FM* locus.

The large number of SNPs showing an allele frequency of 50% in the DUP1 region implies that the complex rearrangement creating the *FM* allele must have involved an inter-chromosomal event involving two different haplotypes creating fixed heterozygosity for the positions in regions of severely suppressed recombination (after the rearrangement) where the two haplotypes differed (Fig. 4C). In conclusion, FM-2 (Fig. 1A) is now the confirmed organization of the *FM* allele and there is strong suppression of recombination for most of the DUP1-inverted DUP2-DUP1 region whereas there is no strong suppression of recombination from the end of the second copy of the DUP1 region to the second copy of DUP2 including the single copy 412.54 kb intervening region (Fig. 4C). This explains the bubble pattern across the DUP1 region (Fig. 4B) in *FM* homozygotes as well as the corresponding pattern of genetic differentiation and nucleotide diversity (Fig. 3C and D).

### Tracing the formation of the *FM* allele

**Fig. 5:**
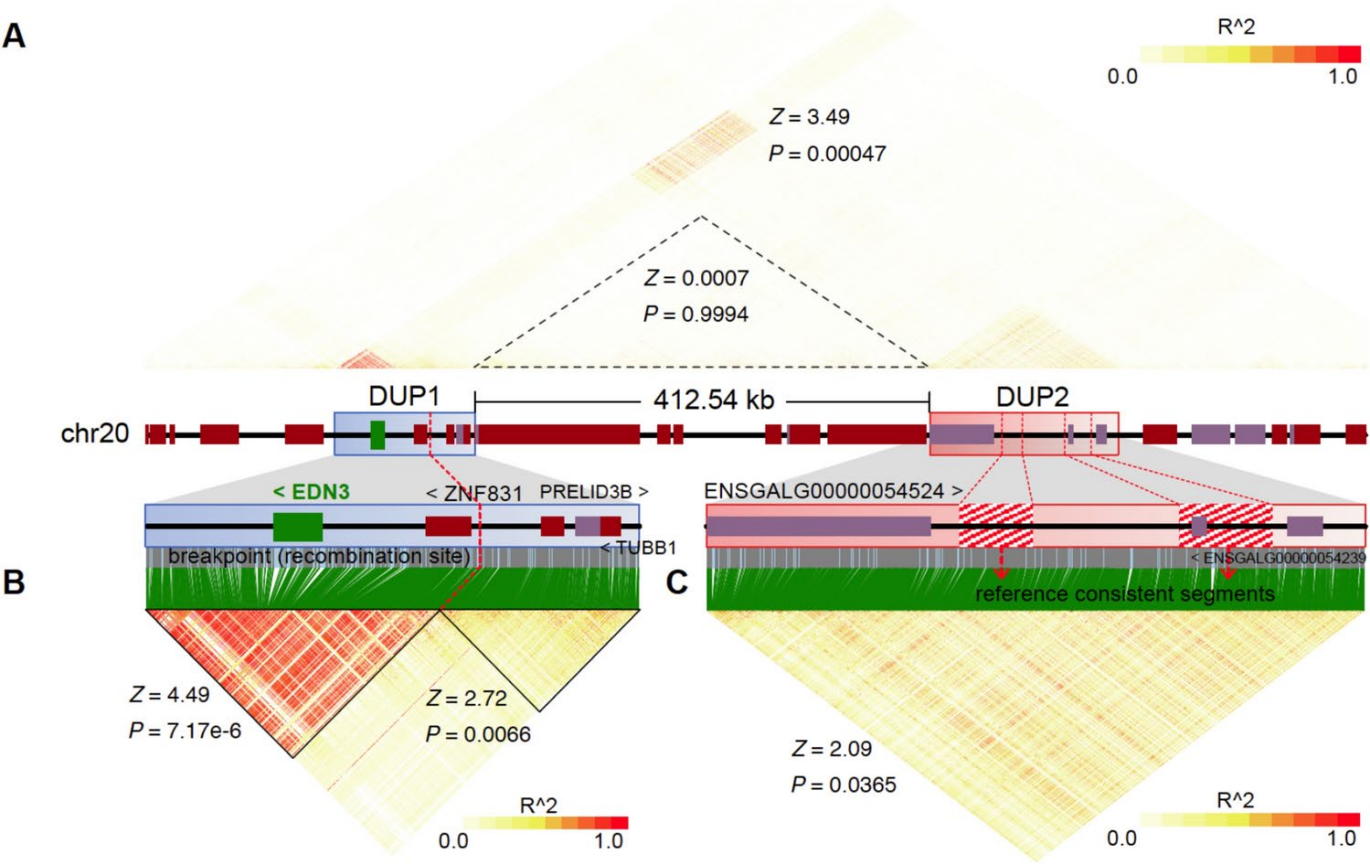
Recombination rate and linkage disequilibrium at the *FM* locus. (**A**–**C**) Linkage disequilibrium analysis of target region (chr20: 10.6–11.7 Mb) (**A**), DUP1 (**B**) and DUP2 (**C**) regions. DUP1 and DUP2 regions are enlarged to show the location of the break in fixed heterozygosity within DUP1 and reference consistent segments (marked with red striped background) within DUP2. Two regions at the FM locus exhibited significantly higher LD retention compared to the genome-wide background (*Z*-scores and *P*-values for different regions are shown).

There is a sharp disruption of the region of fixed heterozygosity within DUP1 in the *FM* allele, marked by a red arrow in Fig. 4B. This could either reflect a recombination with wild-type chromosomes have occurred or a shift from a large region where the two donor haplotypes forming the *FM* duplication showed many sequence differences to a region where they happened to be identical. Similarly, two reference-consistent regions in DUP2 may reflect regions in which one of the copies of DUP2 not undergoing recombination is identical to the genome reference and thus the frequency of the reference allele among chickens homozygous for the *FM* allele will always be 50% or higher (Fig. 4B), because we estimate the allele frequency as the average of sequences from the two copies of DUP2. Linkage disequilibrium (LD) analysis at the *FM* locus also showed a clear LD block boundary between two segments within DUP1 which is in perfect agreement with the break of fixed heterozygosity (Figs. 4B and 5). While LD decay patterns are influenced by recombination rates, population structure, and historical selection events, the significantly elevated LD at the *FM* locus (Fig. 5) are consistent with the presence of an inversion.

## Discussion

### Resolving the structural configuration of the *FM* allele

The *FM* allele constitutes a truly complex structural variant involving two large duplications (127.4 kb and 170.5 kb), an inversion and translocation of the 170.5 duplication and with a 412.5 kb non-duplicated intervening region (Fig. 1). Previous PCR studies established three possible configurations of this complex rearrangement (Fig. 1) that could not be resolved using whole-genome sequencing based on short read data^2,12^. However, one recombinant found in a pedigree analysis provided strong support for the FM-2 configuration^5^. Subsequent studies based on long-read sequencing gave conflicting results, some supporting the FM-1 configuration^10,11^ and others supporting FM-2^8,9,13^. The current study based on extensive whole genome sequence data and the analysis of linkage disequilibrium patterns across more than 40 breeds carrying the *FM* allele now provides conclusive evidence for the FM-2 configuration and that the *FM* allele arose only one time.

The unique feature with an inverted copy of DUP2 inserted between two tandem copies of DUP1 results in strong suppression of recombination explaining the allele frequency bubble at DUP1 when reads from the *FM* allele are aligned to the reference genome (Fig. 4). Furthermore, the data shows that the *FM* mutation must have involved an inter-chromosomal exchange resulting in fixed heterozygosity in the DUP1 region at the sites with sequence differences between the two copies (Fig. 4B). In contrast, fixed heterozygosity and an allele frequency bubble is not noted for DUP2 because one of the DUP2 copies is located outside the region of suppressed recombination (see FM-2 in Fig. 1A). This result is also in complete agreement with the previously reported recombination event between a wild-type and an *FM* allele^2^. Given that the *FM* allele likely originated thousands of years ago^5^, sufficient time has passed for sequence variation in the second copy of DUP2 and the corresponding region of wild-type chromosomes to become randomized due to recombination. This is illustrated by the fact that the nucleotide diversity is clearly reduced only at the DUP1 region and its flanking region (Fig. 3D) consistent with the observed allele frequency bubble (Fig. 4).

### Evolution of the *FM* locus

Our study did not reveal any genetic heterogeneity at the *FM* locus since exactly the same structural rearrangements were detected across all 44 FM chicken breeds analyzed. The *FM* allele must involve multiple structural rearrangements and it is challenging to establish the exact mechanism causing these rearrangements with confidence. However, one possible scenario is that the first event involved a duplication of the entire region from DUP1 to DUP2 (710.4 kb) by unequal cross-over, followed by an inversion involving DUP2 and the intervening sequence (412.5 kb) in which the latter was lost. This scenario is consistent with the fixed heterozygosity for the DUP1 sequence, because the two copies originate from different chromosome homologs. The fixed heterozygosity at DUP1 has since then been maintained due to suppressed recombination while the fixed heterozygosity for DUP2 has been eroded due to recombination affecting the second copy of DUP2 (Fig. 4). It is still an open question whether the two events (the duplication and the inversion) occurred in a single meiosis or in a stepwise fashion over multiple generations.

### Limited capability of long-read data in complex structural variation

For complex structural variations, such as the *FM* locus, which involve copy number variation, rearrangements, inversion, and translocations, long-read sequencing faces limitations for instance when the size of a duplication exceeds the read length. In such cases, the inter-sequence assembly strategy plays a critical role and can significantly influence the accuracy of the assembly^14^. As more and more research focus on large-scale structural variation^15^, such as pan-genome^16^, we suggest that the assembly of regions showing complex structural variation can be validated using population genomics data. Despite previous conflicting reports^2,10–12^, the population genomic approach employed here successfully clarified the structural arrangement, demonstrating the value of population-scale sequencing in deciphering complex genomic rearrangements.

### Structural variants as drivers of phenotypic diversity

The *FM* locus exemplifies how structural variants contribute to phenotypic diversity in domesticated animals^17–22^. These are often gain-of-function mutations with a dominant inheritance, resulting in altered gene expression. Other prominent examples include Dominant white color in pigs^21^, Greying with age in horses^17,18^, and all three comb phenotypes in chicken, Pea-comb^23^, Rose-comb^24^, and Duplex comb^22^. These cases collectively highlight the evolutionary significance of structural variants, particularly in shaping key morphological traits under artificial selection in domestic animals.

## Materials and methods

### Samples collection

In this study, we analyzed 817 chicken samples, comprising 517 FM chicken samples from 44 breeds from various geographic locations worldwide, and an additional 300 wild-type samples (Supplementary Table 1). All samples included in this analysis were previously reported in studies^9–11,25–50^.

### Data processing

Sequencing reads underwent quality control using FastQC (version 0.11.8) to assess read quality metrics. Index adaptors and raw reads with more than 50% bases in low quality (Q ≤ 5) or 10% “N” content were filtered out using Btrim (version 0.3.0)^51^ software. Cleaned reads were then mapped to the reference genome of the red junglefowl (GRCg6a) using the BWA-MEM (version 0.7.17-r1188)^52^ algorithm with default settings. Bam files were sorted using SAMtools (version 1.9)^53^ and PCR duplicates were removed using Picard (version 2.18.6) tools. Sequencing depth relative to the reference genome was calculated using SAMtools (version 1.9)^53^.

The “RealignerTargetCreator” and “IndelRealigner” tools in Genome Analysis Toolkit (GATK, version 3.7)^54^ were used to reduce mismatches around INDELs, with base quality score recalibration (BQSR) performed to reduce mapping errors. Joint calling for SNPs and small INDELs was performed using the “HaplotypeCaller” and “GenotypeGVCFs” tools from GATK (version 3.7)^54^. Low quality SNPs were filtered, with parameters following previous research^7,55^: “QUAL < 30.0 || QD < 2.0 || MQ < 40.0 || FS > 60.0 || MQRankSum < − 12.5 || ReadPosRankSum < − 8.0 || SOR > 3.0”. Loci with max-missing rate above 0.10 and minor allele frequency less than 0.05 (–maf 0.05; –max-missing 0.9) were filtered using VCFtools (version 0.1.16)^56^. Only biallelic loci were retained.

### SVs identification

SVs were detected using relative sequence depth analysis. Sequence depth was calculated using SAMtools (version 1.9)^53^ to identify the genomic regions of interest, specifically focusing on the DUP1 (chr20:10,766,772–10,894,151) and DUP2 (chr20:11,306,686–11,477,501) regions. The relative sequencing depth is the ratio of the sequencing depth of the candidate region to the whole genome background. To differentiate between wild type (FM*N/*N*), homozygous (FM*FM/*FM*), and heterozygous (FM*FM/*N*) samples, relative sequence depth analysis was performed using the average sequence depth of the target region divided by the average sequence depth of whole genome or chromosome 20.

### Nucleotide diversity and genetic differentiation

Nucleotide diversity (π) and genetic differentiation (*F*_ST_) was calculated using VCFtools (version 0.1.16)^56^ using a 20 kb window length with 10 kb sliding window, focusing on the SV regions and their flanking sequences (chr20:10,600,000–11,700,000). To evaluate the statistical significance of *F*_ST_ estimates across different genomic regions, we performed a *Z*-score transformation relative to the genome-wide distribution. *P*-values were derived from the standard normal distribution, and Benjamini–Hochberg false discovery rate (FDR) correction was applied to control for multiple testing. *F*_ST_ windows with FDR-adjusted *P*-values < 0.05 were considered significantly different from the genome-wide expectation. Allele frequency distributions were analyzed using VCFtools (version 0.1.16)^56^, with particular attention to differences between wild type, heterozygous, and homozygous FM chickens. Regions showing significant deviations in allele frequency were identified and further examined for recombination breakpoints manually.

### Phylogenetic analysis

Phylogenetic relationships among FM chickens were inferred using the identified SV regions. Neighbor-joining (NJ) trees were constructed using and RapidNJ software and visualized using iTOL^57^. Separate trees were constructed for the DUP1 and DUP2 regions.

### Linkage disequilibrium analysis

To assess LD decay patterns across the FM locus, we computed pairwise linkage disequilibrium (LD) using r^2^ between SNPs using LDBlockShow (version 1.40)^58^ with default parameters. LD decay patterns were used to infer historical recombination rates, assuming a constant recombination landscape over time. The LD block structure was analyzed to identify boundaries within the DUP1 and DUP2 regions, indicating possible recombination breakpoints. To determine whether LD decay in the *FM* locus was significantly different from the genome-wide background, we performed a genome-wide *Z*-score transformation within 10 kb windows. We then converted *Z*-scores to *P*-values using the standard normal distribution and applied FDR correction to control for multiple testing. Regions with FDR-adjusted *P*-values < 0.05 were considered significantly different from genome-wide LD expectations. This analysis assumes that LD patterns reflect both historical recombination events and population demographic history. Specifically, LD tends to persist in regions under strong selection, low recombination, or with recent population bottlenecks.

## Supplementary Information

Supplementary Information 1.

Supplementary Information 2.

## Data Availability

All of the whole genome sequencing (WGS) datasets used in this study have been previously reported (Supplementary Table 1). The variant call format (VCF) data was deposited in the Genome Variation Map (GVM) under accession project number PRJCA032154.

## Code availability

All analyses were performed using previously published or developed tools, as indicated in Methods. No custom code was developed or used.
