# Supplementary material for: Population genomic analysis identifies the complex structural variation at the fibromelanosis (FM) locus in chicken
Source: Sci Rep. 2025 Mar 18;15:9239. doi: 10.1038/s41598-025-94250-4 (PMC11920206; doi:10.1038/s41598-025-94250-4)
Supplement: Supplementary file 1 — Supplementary Information 1. [file 41598_2025_94250_MOESM1_ESM.pdf]

Supplementary figures

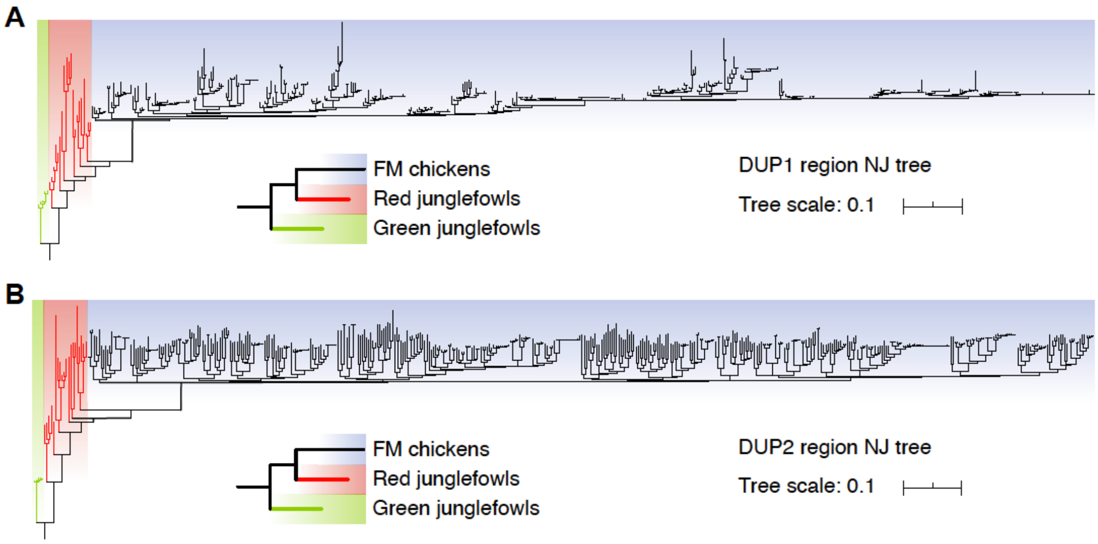

Supplementary Fig. 1: Phylogenetic analysis of the *FM* locus.

A Phylogenetic tree of the DUP1 region. Short branch lengths for most individuals indicate a high level of genetic identity, with some longer branch lengths likely resulting from limited recombination events. Different clades are highlighted with background colors: green for the outgroup, green junglefowls (*FM*N/N*), red for red junglefowls (*FM*N/N*), blue for FM chickens (*FM*FM/FM*). **B** Phylogenetic tree of the DUP2 region. Normal branch lengths are observed in the DUP2 region, contrasting with the shorter branch lengths seen in the DUP1 region, consistent with the FM-2 configuration in Fig. 1A.
